# Mapping of Novel Leaf Rust and Stem Rust Resistance Genes in the Portuguese Durum Wheat Landrace PI 192051

**DOI:** 10.1534/g3.119.400292

**Published:** 2019-07-05

**Authors:** Meriem Aoun, James A. Kolmer, Matthew N. Rouse, Elias M. Elias, Matthew Breiland, Worku Denbel Bulbula, Shiaoman Chao, Maricelis Acevedo

**Affiliations:** * Department of Plant Pathology, North Dakota State University, Fargo, ND, 58108,; † United States Department of Agriculture–Agricultural Research Service (USDA-ARS), Cereal Disease Laboratory, and Department of Plant Pathology, University of Minnesota, St. Paul 55108,; ‡ Department of Plant Sciences, North Dakota State University, Fargo, ND, 58108,; § Ethiopian Institute of Agricultural Research, P.O. Box 32, Debre Zeit, Ethiopia,; ** SDA-ARS Cereal Crops Research Unit, Fargo, ND, 58102, and; †† International Programs, College of Agriculture and Life Sciences, Cornell University, Ithaca, NY, 14853

**Keywords:** DGGW, disease resistance, Ethiopia, food security, leaf rust, new resistance, Puccinia, QTL, SNP, wheat

## Abstract

Leaf rust caused by *Puccinia triticina* Erikss. (*Pt*) and stem rust caused by *Puccinia graminis* f. sp. *tritic*i Erikss. & E. Henn (*Pgt*) are serious constraints to production of durum wheat (*Triticum turgidum* L). The objective of this study was to identify leaf rust resistance (*Lr*) and stem rust resistance (*Sr*) genes/QTL in Portuguese durum landrace PI 192051. Four *Pt*-isolates, representing three virulence phenotypes (BBBQJ, BBBSJ & EEEEE) and six *Pgt*-races TTKSK, JRCQC, TKTTF, QFCFC, TPMKC and TMLKC were used to evaluate 180 recombinant inbred lines (RILs) derived from the cross Rusty (rust susceptible) × PI 192051-1 (rust resistant) at the seedling stage. The RILs were also phenotyped at the adult-plant stage in a stem rust nursery in Ethiopia in 2017. The RILs were genotyped using the Illumina iSelect 9K wheat SNP array. PI 192051-1 carries a previously unidentified major *Sr* gene designated as *QSr.ace-7A* on chromosome arm 7AS and *Lr* gene *Lr.ace-4A* in the pericentromeric region of chromosome 4A. In addition, three minor *Sr* QTL *QSr.ace-1A*, *QSr.ace-2B* and *QSr.ace-4A* were mapped in PI 192051-1 on chromosomes 1AL, 2BL, and 4A, respectively *Lr.ace-4A* could be co-located or tightly linked to *QSr.ace-4A*. Markers linked to the identified QTL/genes can be used for marker assisted selection. These findings enrich the genetic basis of rust resistance in both durum and common wheat.

Durum wheat (*Triticum turgidum* L. var. *durum* (Desf.); 2n = 4x = 28, AABB) is an important cereal crop grown and consumed mainly in the Mediterranean basin (Northern Africa and Southern Europe). Other durum wheat producing countries include Canada, Mexico, USA, Australia, and Ethiopia. Durum wheat represents 5–10% of total global wheat production and is important for food security in small geographical regions in developing countries (Ammar *et al.* 2006).

Leaf rust caused by *Puccinia triticina* Erikss. (*Pt*) and stem rust caused by *Puccinia graminis Pers.:Pers*.f. sp. *tritici* Erikss. & E. Henn (*Pgt*) are serious threats to durum wheat production. Leaf rust is widespread throughout durum growing areas in Mexico, USA, India, Ethiopia, and the entire Mediterranean basin (Singh *et al.* 2004; Goyeau *et al.* 2006, 2012; Kolmer 2013, 2015; Mishra *et al.* 2015; Kolmer and Acevedo 2016). The *Pt* races isolated from durum are often different from those prevalent on hexaploid common wheat (*T. aestivum* L.; 2n = 6x = 42, AABBDD) and often exhibit avirulence on many of the commonly reported leaf rust resistance (*Lr*) genes in common wheat (Huerta-Espino and Roelfs 1992; Ordoñez and Kolmer 2007a). *Pt* isolates collected in durum fields in several countries share similar phenotypic reactions on the ‘Thatcher’ near-isogenic lines and similar or identical SSR genotype, suggesting a common origin (Ordoñez and Kolmer 2007a, 2007b). However, some isolates collected in Ethiopia (designated as race EEEEE) with virulence on durum wheat are avirulent to Thatcher and have a distinct SSR genotype compared to *Pt*-isolates from durum collected worldwide (Huerta-Espino and Roelfs 1992; Ordoñez and Kolmer 2007a, 2007b; Kolmer and Acevedo 2016; Aoun *et al.* 2018).

A few cataloged *Lr* genes (*Lr3a*, *Lr14a*, *Lr27*+*Lr31*, *Lr61*, *Lr72*, and *LrCamayo*) have been reported in durum wheat cultivars (Herrera-Foessel *et al.* 2007, 2008a, 2008b 2014; Huerta-Espino *et al.* 2009a). In addition, the majority of the durum cultivars grown globally appear to carry single race-specific resistance genes. Consequently, this has favored selection for *Pt* races with virulence to all these genes in just a few years after their deployment (Ordoñez and Kolmer 2007a; Huerta-Espino *et al.* 2009a, 2009b 2011; Goyeau *et al.* 2012; Herrera-Foessel *et al.* 2014). The large majority of durum cultivars are susceptible to the durum type *Pt* races. Therefore, there is an urgent need to identify new *Lr* genes in durum wheat.

Stem rust is a historically devastating disease of common and durum wheat. *Pgt* race TTKSK (Ug99) and its evolving variants have virulence to several widely used wheat stem rust resistance (*Sr*) genes (Jin *et al.* 2007; Singh *et al.* 2011). Currently, more than 70 *Sr* genes are characterized in wheat. Approximately 31 genes continue to be effective against at least one race of the Ug99 lineage (Singh *et al.* 2011, 2015; Rouse *et al.* 2011, 2014a). Around half of these genes were introgressed from wild wheat relatives (Rouse *et al.* 2014a; Singh *et al.* 2015) and only a few genes have been mapped in durum wheat. Resistance to the Ug99 lineage in North American durum cultivars is mainly due to *Sr13* alleles, of which *Sr13a* was first identified in Khapstein, a bread wheat derivative of emmer (*T*. *turgidum*, L. ssp. *dicoccum*) cv. Khapli (Knott 1962, Jin *et al.* 2007, Klindworth *et al.* 2007, Zhang *et al.* 2017). A possible *Sr8* allele designated as *Sr8155B1* is another resistance source in durum wheat found to confer resistance to a variant of the Ug99 race group TTKST, but not to race TTKSK (Nirmala *et al.* 2017). Besides the limited number of characterized *Sr* genes/alleles in durum wheat cultivars, the continuous emergence of virulent *Pgt*-races including TRTTF, TTRTF, JRCQC, and TKTTF that do not belong to the Ug99 lineage have been reported (Olivera *et al.* 2012, 2015; rusttracker.cimmyt.org 2017). The recent stem rust outbreak in Sicily, Italy in 2016 caused by race TTRTF, which is unrelated to the Ug99 group, represents a serious threat to the common wheat and durum production in the Mediterranean basin (rusttracker.cimmyt.org 2017).

Landraces may carry new sources of resistance that can be exploited to enrich the narrow resistance spectrum currently found in adapted cultivars. The use of landraces is limited in most breeding programs (Bonman *et al.* 2007; Newton *et al.* 2010; Bux *et al.* 2012; Gurung *et al.* 2014), which has created a diversity bottleneck resulting in lack of disease resistance (Tanksley and McCouch 1997). Use of landraces like ‘PI 192051’ from the USDA-National Small Grains Collection (NSGC) could contribute to enhanced rust resistance in durum wheat. PI 192051 was found to be highly resistant to several *Pt* races (Aoun *et al.* 2016, 2017) and *Pgt*-races (Olivera *et al.* 2012; Chao *et al.* 2017).

Aoun *et al.* (2017) reported that F_1_ plants of the cross Rusty × PI 192051-1 were resistant to *Pt*-race BBBQJ collected from durum wheat in California. This *Pt* race has a similar virulence pattern to the Mexican race BBG/BN that caused the leaf rust outbreak in Northwestern Mexico in 2001 (Singh *et al.* 2004; Kolmer 2015). Segregation of F_2:3_ families indicated the presence of a single gene conferring resistance to race BBBQJ. The objective of the present study was to map leaf rust and stem rust resistance genes/QTL in PI 192051 and to develop SNP markers tightly linked to the identified loci.

## Materials and methods

### Population development

The population used in this study was developed by crossing ‘Rusty’ with a single plant selection of PI 192051, PI 192051-1 (Aoun *et al.* 2017). Rusty (Reg. no. GS-155, PI 639869), a durum accession that is a near universally susceptible to wheat stem rust, was selected and released in 2004 by the USDA-ARS Northern Crops Science Laboratory, Fargo, ND, and North Dakota State University (Klindworth *et al.* 2006). Durum wheat landrace PI 192051 from Lisboa, Portugal where it is known as ‘Amarelo de Barba Branca’ was deposited in the NSGC in 1950. In this study, Rusty was the susceptible genotype and PI 192051-1 was resistant to both leaf rust and stem rust (Table 1). The hybrid population was advanced by single seed descent at North Dakota Agricultural Experiment Station Greenhouse Complex, Fargo, ND, USA and 180 F_6_ RILs were produced.

**Table 1:** Reactions of PI 192051-1 and Rusty to leaf rust and stem rust

Race/Experiment	Origin	Median reaction of PI 192051-1	Median reaction of Rusty
***Pt* race: Seedling stage**			
BBBQJ_CA1.2	USA/California	0;	3+
BBBQJ_Mor38-2	Morocco	;	3
EEEEE_Eth50-4	Ethiopia	0;	33+
BBBSJ_Tun20-4	Tunisia	;	3
***Pgt* race: Seedling stage**			
QFCFC	USA/North Dakota	2	33+
TPMKC	USA/North Dakota	2	33+
TMLKC	USA/North Dakota	2	33+
JRCQC	Ethiopia	2	3+
TKTTF	Ethiopia	22+	3+
TTKSK	Kenya	2-	33+
***Pgt* race: Adult plant stage**			
Eth2017_1	Ethiopia	5 MS	30 S
Eth2017_2	Ethiopia	20 MSMR	50 SMS

### Leaf rust evaluation

The F_6_-RILs were screened at the seedling stage with four *Pt*-isolates virulent on durum wheat in the biosafety level-2 facility at the Fargo Agricultural Experiment Station Greenhouse Complex. *Pt* isolates collected from Ethiopia, Morocco, Tunisia, and USA (California) were designated as Eth50-4, Mor38-2, Tun20-4, and CA1.2, respectively. The virulence/avirulence phenotypes of these isolates were based on seedling stage infection types using 20 Thatcher near-isogenic lines (NILs) as described by Long and Kolmer (1989). Both CA1.2 and Mor38-2 were race BBBQJ (virulent to genes *LrB*, *Lr10*, *Lr14b*, and *Lr20*), and Tun20-4 was race BBBSJ (virulent to *LrB*, *Lr10*, *Lr14a*, *Lr14b*, *Lr20*). Isolate Eth50-4 was avirulent on seedlings of the cultivar Thatcher and was given the race designation EEEEE. The RILs were evaluated for leaf rust response in twice replicated randomized complete blocks (RCBD) with exception of the experiment for isolate CA1.2 that was replicated thrice. In each replicate 8–10 plants/RIL were tested. The parents of the cross, the common wheat cultivar Thatcher and the leaf rust susceptible durum line ‘RL6089’ were included in each 50-cell tray as checks. Two replicates of Thatcher NIL differentials were included in each experiment to confirm the virulence phenotype of *Pt*-isolates. The seedlings were grown under the same greenhouse conditions as described by Kertho *et al.* (2015). Inoculum increase, inoculation process, and greenhouse conditions under which the inoculated plants were grown until disease screening were as described by Aoun *et al.* (2016).

Leaf rust infection types (ITs) were evaluated 12–14 days after inoculation on the second leaf using a 0–4 scale (Long and Kolmer 1989; McIntosh *et al.* 1995), where IT 0 = no visible symptom, ; = hypersensitive flecks, 1 = small uredinia with necrosis, 2 = small- to medium-size uredinia surrounded by chlorosis, 3 = medium-size uredinia with no chlorosis or necrosis, and 4 = large uredinia with no necrosis or chlorosis. Larger and smaller uredinia than expected for each IT were designated with + and −, respectively. Seedlings showing ITs of 0 – 2^+^ and ‘X’ (a mixture of low and high ITs evenly distributed on the leaf surface) were considered resistant, whereas seedlings showing ITs of 3–4 were considered susceptible (Long and Kolmer 1989; McIntosh *et al.* 1995). The RILs that showed only resistant plants across replicates were considered homozygous resistant (HR) and the RILs that showed only susceptible plants across replicates were considered homozygous susceptible (HS). RILs that showed both resistant and susceptible plants were classified as segregating (Seg).

### Stem rust evaluation

The RILs were also tested at seedling stage with African *Pgt*-races TTKSK (isolate # 04KEN156/04), TKTTF (isolate # 13ETH18-1) and JRCQC (isolate # 08ETH03-1) and with the North American *Pgt* races QFCFC (isolate # 370C), TPMKC (isolate # TNMKsp1) and TMLKC (isolate # 72-41-sp2). For African *Pgt*-races, F_6_-RILs were phenotyped in the biosafety level-3 facility at the USDA-ARS Cereal Disease Laboratory in St. Paul, MN, whereas for the North American races, F_7_-RILs were phenotyped at the Agricultural Experiment Station Greenhouse Complex at North Dakota State University in Fargo, ND. The RILs were planted in a RCBD with two replicates (five seedlings/RIL/replicate) for all six *Pgt* races. Inoculation conditions were similar at both St. Paul and Fargo.

Urediniospores stored at -80° were heat shocked at 45° for 15 min, then rehydrated at 80% relative humidity created with a KOH solution for 2–4 h under room temperature (Rowell 1984). The spores were then suspended in mineral oil (Sotrol 170, Phillips Petroleum, Borger, TX, USA), then sprayed onto the primary leaves of the seedlings. The inoculated seedlings were placed in a humidity chamber in darkness for 14–16 h at 18°, then retained for 3–4 h under florescent light to enhance spore germination. The plants were then placed in the greenhouse at 18 ± 2° and 16 h photoperiod for 10–12 days when ITs were scored using the Stakman 0 – 4 scale (Stakman *et al.* 1962). Plants showing ITs 0 – 2^+^3 were considered resistant and those with IT of 3 – 4 were considered susceptible. The classification of RILs into HR, HS, and Seg was conducted as described for leaf rust.

For both leaf rust and stem rust data, the χ2 test for goodness-of-fit at 95% level of confidence was used to assess the deviation of observed segregation from 1HR: 1HS theoretically expected of RILs at F_6_ and F_7_ generations.

Field evaluation of the RIL population at the adult-plant stage was carried out at the international stem rust nursery at the Ethiopian Institute for Agricultural Research Center in Debre Zeit (EIAR-DZ) in 2017. This nursery is an international durum wheat screening site for stem rust as part of the Borlaug Global Rust Initiative’s Durable Rust Resistance in Wheat Project. The station is located at 1,900 m above sea level at 8° 44’ N latitude and 38° 85’ E longitude. This center is a hotspot for wheat stem rust and can be used for two cropping seasons (July–November and January–May) (Letta *et al.* 2013).

A total of 160 F_7_-RILs were phenotyped for stem rust response in Ethiopia in 2017. Rusty, PI 192051-1 and the local check ‘Arendeto’ were included four times (every after 40 entries). The RILs, parents and Arendeto were planted in hill plots with 20–30 seeds/RIL. Stem rust spreaders of susceptible wheat cultivars were artificially inoculated 2-3 times with races TTKSK (Ug99) and JRCQC starting from stem elongation stage. Other races such as TKTTF, TRTTF, RRTTF that are known to be present in this region (Olivera *et al.* 2012, 2015) may have been present in the nursery.

Stem rust severity and response were assessed twice at the soft-dough stage of plant development on June 8^th^ and June 23^rd^, 2017. Disease severities were scored following a modified Cobb scale (Peterson *et al.* 1948; Roelfs *et al.* 1992). The lines were classified, based on the host response into resistant (R), moderately resistant (MR), intermediate (M), moderately susceptible (MS), and susceptible (S) as described by Roelfs *et al.* (1992). A combination of two categories of host response on the same line was possible.

### SNP genotyping and linkage mapping

Leaf tissue from Rusty, PI 192051-1, and 180 F_6_-RILs were collected, lyophilized for 24 h, and ground as described by Rouse *et al.* (2012). The DNA was extracted using the CTAB protocol (Riede and Anderson 1996) modified by Liu *et al.* (2006). The DNA was diluted to 50 ng/µl and genotyped at the USDA-ARS Small Grain Genotyping Lab at Fargo using the Illumina iSelect 9K wheat SNP array (Cavanagh *et al.* 2013) following the manufacturer’s protocol (Illumina Inc., SanDiego, CA, U.S.A.). The SNP genotypes were manually scored using version 1.0 of the polyploid clustering module for Illumina GenomeStudio version 1.9.4 (Illumina Inc.).

Markers were sorted according to the parents and all monomorphic markers were removed. Heterozygous genotypes were converted into missing data and polymorphic markers with <20% missing data were used for mapping. The maps were generated using MapDisto version 1.7.7.0.1.1 (Lorieux 2012) with a LOD score of 4 and maximum recombination frequency of 0.3. The Kosambi mapping function (Kosambi 1943) was used to convert recombination frequencies between SNPs into map distances in centiMorgans (cM). The robustness of the map was evaluated using “ripple order” and “check inversions” commands. Problematic loci that increased the map length by more than 4 cM were identified using the “drop locus” command. After removing each problematic marker, the ripple order and check inversion commands were applied to revalidate the robustness of the maps. The developed linkage maps covered all 14 tetraploid wheat chromosomes and the linkage groups were assigned to chromosomes by comparison with the tetraploid wheat consensus map (Maccaferri *et al.* 2015).

### QTL analysis

The seedling rust responses of the RILs were converted from the 0–4 Stakman scale to a 0–9 linear scale as described by Zhang *et al.* (2014). In the case of segregating RILs, the weighted means were calculated, where the weighted mean of the RIL= (the most predominant linearized IT *2 + the least predominant linearized IT)/3. The means of the replicates were used for quantitative trait locus (QTL) mapping. The stem rust response data at adult-plant stage were converted into coefficient of infection (CI) and then used for QTL analysis. The CI was obtained by multiplying the severity and a constant for host response, where immune = 0.0, R = 0.2, MR = 0.4, MS = 0.8, S = 1.0, RMR = 0.3, MRMS= 0.6 and MSS = 0.9 (Yu *et al.* 2011). Pairwise correlations between traits were calculated and plotted on R 3.4.1 (R Core Team 2016) using the ‘corrplot’ package (Wei 2013). Correlation values were considered significantly different from zero at *P* value ≤ 0.05.

QTL analysis was conducted using QGene 4.0 (Joehanes and Nelson 2008) using three QTL analysis methods: composite interval mapping (CIM), multiple interval mapping (MIM) and MIM-based on a general linearized framework (MIM-GLZ). For CIM and MIM, the QTL analysis was based on transformed phenotypic data (Ln (1+ y) where y= phenotypic response. For MIM-GLZ, the QTL analysis was performed on the non-transformed data.

The significant LOD threshold values were determined by performing permutation tests (1000 iterations) at an experiment-wide error of α= 0.05 and the coefficient of determinations (R^2^) were calculated and used to determine the amount of phenotypic variation explained by the QTL. The 95% confidence intervals of QTL were estimated using the 2-LOD drop method as described by Lander and Botstein (1989). QTL were considered co-localized if their confidence intervals overlapped. After identifying the positions of the QTL on specific linkage groups, co-segregating SNPs were excluded to reduce marker redundancy of the maps. SNP marker flanking sequences were BLAST against the Chinese Spring wheat genome sequence (IWGSC_RefSeq_v1.0) to identify the physical locations of the SNP markers linked to the identified QTL.

### Data Availability

The RILs of the population Rusty × PI 192051-1 are available upon request. Table S1 contains phenotypic data used in this study and Table S2 contains SNP data of the RILs. Supplementary materials were uploaded to Figshare. The authors affirm that all data necessary for confirming the conclusions of the article are present within the article, figures, and tables. Supplemental material available at FigShare: https://doi.org/10.25387/g3.8428898.

## Results

### Leaf rust response

**Figure 1:**
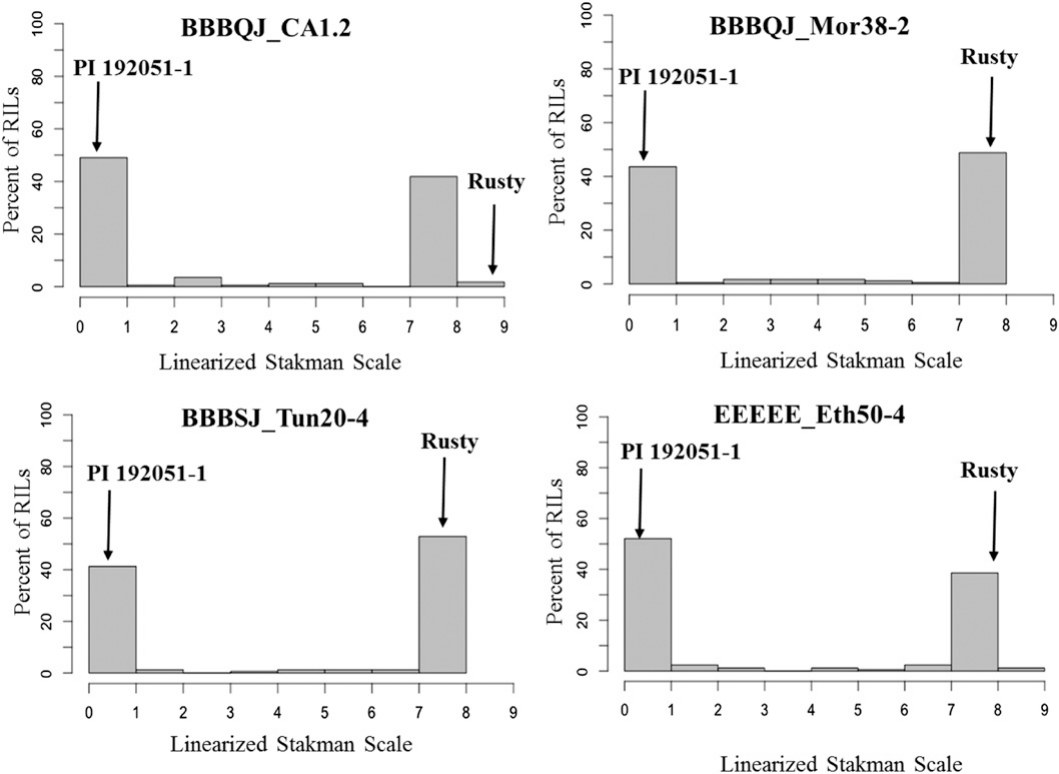
Distributions of the seedling response data for *Puccinia tritici*na isolates BBBQJ_CA1.2, BBBQJ_Mor38-2, BBBSJ_Tun20-4, and EEEE_Eth50-4 for recombinant inbred lines (RILs) from the cross Rusty × PI 192051-1. Distribution is expressed as the percent individuals within a linearized Stakman scale (0-9). Median phenotypes for PI 192051-1 and Rusty are indicated on the graph.

Evaluation of 155–172 F_6_-RILs for response to the four *Pt* isolates showed bimodal distributions of ITs ranging from 0; to 3^+^. The lowest (‘0;’) and highest (‘3^+^’) ITs corresponded to those of the resistant and susceptible parents, respectively (Figure 1 and Table 1). Grouping the RILs into HR and HS showed a segregation ratio of 1HR: 1HS based on χ2 test (*P* ≥ 0.05) for all four *Pt* isolates (Table 2 and Table S1). This suggests that the leaf rust resistance to each race in PI 192051-1 was conferred by a single gene. Small differences in number of HR and HS RILs in the four tests were due to differences in numbers of RILs tested with each *Pt* isolate (Table 2 and Table S1). There were significant correlations (Pearson correlation coefficients (r) were 0.9 to 1, *P* value <0.05) between ITs of the RILs for the four studied *Pt* isolates, suggesting that the same gene conferred resistance to these *Pt* isolates (Figure S1).

**Table 2:** Frequencies of homozygous resistant, homozygous susceptible and heterozygous RILs derived from Rusty × PI 192051-1 when tested at the seedling stage with four *P. triticina* (*Pt*) isolates

*Pt* race	Homozygous resistant	Segregating	Homozygous susceptible	Total	*P* value for χ^2^_1HR:1HS_
BBBQJ_CA1.2	85	9	73	167	0.2 ^ns^
BBBQJ_Mor38-2	80	7	85	172	0.72 ^ns^
EEEEE_Eth50-4	91	5	67	163	0.16 ^ns^
BBBSJ_Tun20-4	67	5	83	155	0.43 ^ns^

ns: observed ratio of homozygous resistant (HR) and homozygous susceptible (HS) RILs is not significantly different than the ratio 1HR: 1HS at 95% level of confidence.

### Stem rust phenotypic evaluation

**Figure 2:**
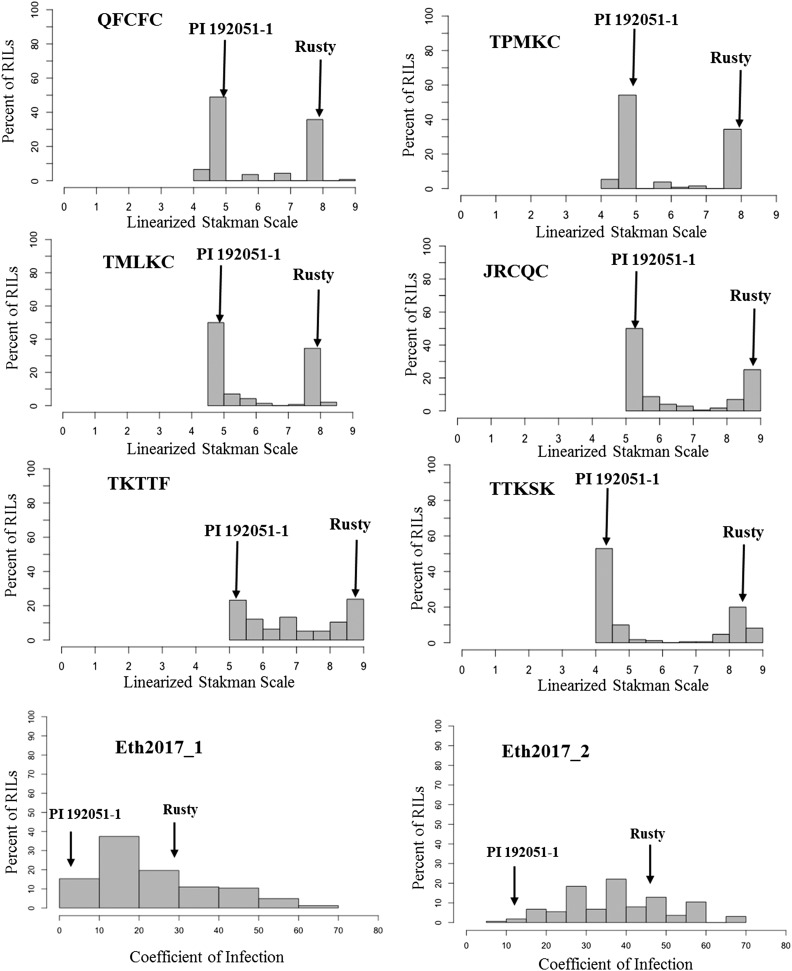
Distributions of the seedling data to *Puccinia graminis* f. sp. *tritici* races QFCFC, TPMKC, TMLKC, JRCQC, TKTTF, and TTKSK and field data in Ethiopia 2017 at two scoring dates (2017-1 and 2017-2) for recombinant inbred lines (RILs) of the cross Rusty × PI 192051-1. Median phenotypes for PI 192051-1 and Rusty are indicated on the graph. X- axes correspond to linearized Stakman scale (0 – 9) for the seedling data and coefficient of infection for the adult plant stage data.

For stem rust seedling tests, 137–172 RILs were evaluated with three US races and three African races (Table 1). The median ITs of the resistant parent PI 192051-1 to African *Pgt* races TTKSK, JRCQC and TKTTF were ‘2^-^’, ‘2’, ‘22^+^’, respectively, whereas the median IT of PI 192051-1 was ‘2’ to all the three US races. For all the *Pgt* races, the lowest and highest IT observed in the RILs were similar to those of the resistant parent PI 192051-1 and susceptible parent Rusty, respectively (Figure 2 and Table 1). The pattern of segregation of RILs screened did not fit a ratio expected for a single gene (based on χ2 test at 95% level of confidence) except for *Pgt*-race QFCFC (Table 3 and Table S1). We observed 35 and 23 segregating families to *Pgt* races JRCQC and TKTTF, respectively, which is higher than the expected number of segregating RILs at the F_6_ generation. This deviation is likely caused by the intermediate nature of the resistant reactions (ITs 22+ to 2+3) to races JRCQC and TKTTF. The complex intermediate reactions make classification of homozygous *vs.* segregating families difficult.

**Table 3:** Frequencies of homozygous resistant, homozygous susceptible and heterozygous RILs derived from Rusty × PI 192051-1 when tested at the seedling stage with six *P. graminis* f. sp. *tritici (Pgt)* races

*Pgt* race	Homozygous resistant	Segregating	Homozygous susceptible	Total	*P* value for χ^2^_1HR:1HS_
QFCFC	81	1	55	137	0.06 ^ns^
TPMKC	79	3	49	131	0.02*
TMLKC	84	5	53	142	0.005**
JRCQC	91	23	58	172	< 0.00001***
TKTTF	73	35	64	172	< 0.00001***
TTKSK	107	7	56	170	0.0003***

ns: observed ratio of homozygous resistant (HR) and homozygous susceptible (HS) RILs is not significantly different than the ratio 1HR: 1HS; *, **, ***: Observed ratio of HR and HS RILs is significantly different from the ratio 1HR: 1HS at 95%, 99%, and 99.9% level of confidence, respectively.

There were significant correlations between the RIL responses to the six *Pgt* races (0.6 ≤ r ≤ 0.9, *P* < 0.05) (Figure S1).

The RILs were evaluated in two consecutive scoring dates (Eth2017_1 and Eth2017_2) in the field nursery in Ethiopia (Eth2017), The median infection responses of Rusty were 30S and 50SMS whereas those of PI 1902051-1 were 5MS and 20MSMR at Eth2017_1 and Eth2017_2, respectively. Arendeto showed median disease responses of 30MSS and 40S at Eth2017_1 and Eth2017_2, respectively. Transgressive segregation led to responses higher than that of Rusty (Figure 2, Table1, and Table S1). There was high correlation between the RIL data obtained at the two scoring dates (r = 0.7, *P < 0.05*). Responses to *Pgt* races at the seedling stage were significantly correlated (0.4 ≤ r ≤ 0.6, *P* < 0.05) with the Eth2017 adult-plant stage data. RIL seedling responses to *Pgt* races QFCFC, TMLKC, and TKTTF had relatively low but significant correlations (r = 0.2, *P < 0.05*) with RIL responses to *Pt* isolates (Figure S1).

### Linkage mapping

A total of 1,138 polymorphic SNPs with <20% missing data were identified using Illumina’s iSelect 9K SNP array. These markers were used to generate a genetic map with a total length of 1,436.24 cM distributed across 20 linkage groups covering the 14 chromosomes of the tetraploid wheat. The number of markers per linkage group varied from 16 SNPs (seven unique loci) in linkage group 2A-1 to 107 SNPs (56 unique loci) in linkage group 5B. The lengths of the linkage groups ranged from 2.8 cM in linkage group 7A-1 to 150.4 cM in linkage group 5B. A total of 176 SNPs showed evidence of segregation distortion from 1:1 ratio based on χ2 goodness of fit test at 99% level of confidence. Majority of the distorted markers were located on the linkage groups 2B (34 markers), 3B-2 (49 markers) and 6B (69 markers). For the remaining linkage groups, there number of distorted markers ranged from zero to six SNPs (Figure S2, Table S3).

### Lr gene mapping

Leaf rust phenotypic data for all the four *Pt* isolates showed bimodal distributions and the segregation ratio were not significantly different from 1HR: 1HS based on the χ2 test for goodness-of-fit at 95% level of confidence (Figure 1 and Table 2). The phenotypic data were converted into binary data based on classification as HR or HS. The binary phenotypic data were then merged with the genotypic data for linkage mapping using MapDisto. QTL analysis was also performed on the linearized leaf rust scores (0– 9 scale).

**Figure 3:**
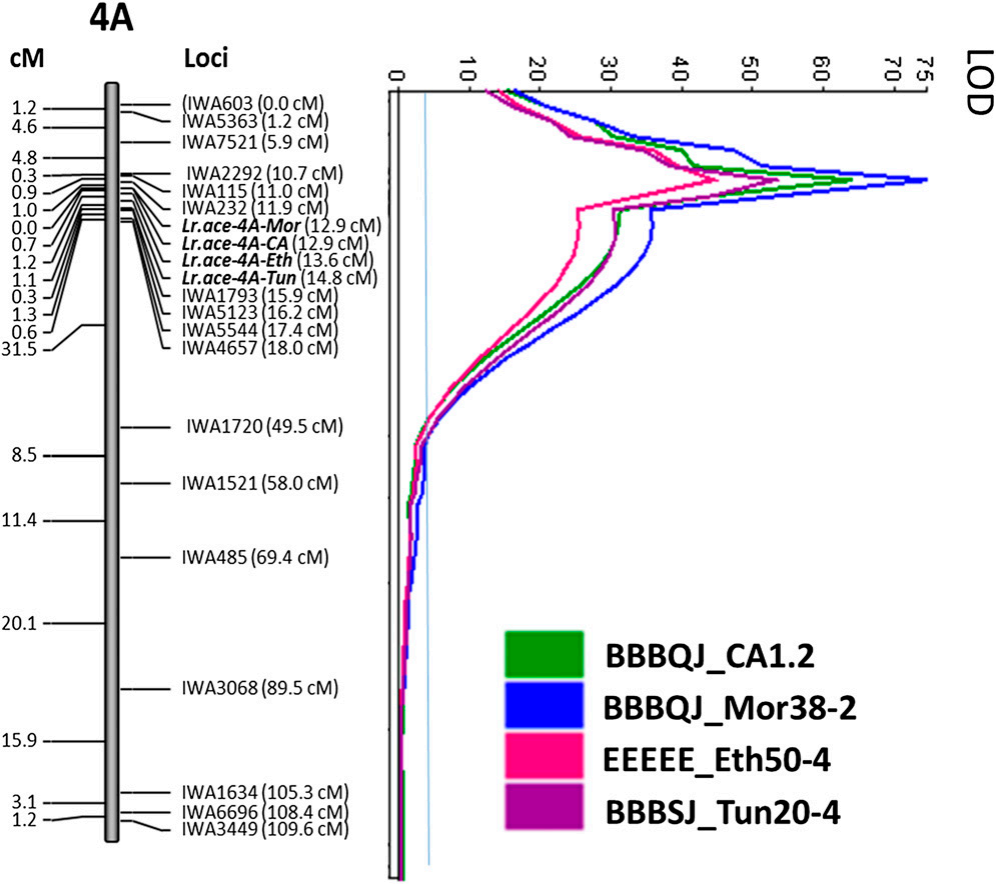
Mapping of leaf rust resistance gene *Lr.ace-4A* in PI 192051-1 to *P. triticina* isolates BBBQJ_CA, BBBQJ_Mor, BBBSJ_Tun, and EEEEE_Eth at seedling stage. QTL analysis was performed using composite interval mapping. The QTL analysis LOD threshold is indicated with the blue line. Co-segregating markers were excluded from this map. SNPs and their cosegregating markers are presented in Figure S2.

The *Lr* gene in PI 192051-1 conferring resistance to the four *Pt* isolates was mapped to chromosome 4A. The *Lr* gene region was delimited by *IWA232* and *IWA1793* (11.9– 15.9 cM) covering a 4.0 cM region (Figure 3 and Table 4). *Lr.ace-4A-CA* (to race BBBQJ_CA1.2) and *Lr.ace-4A-Mor* (to race BBBQJ_ Mor38-2) were mapped to the same genomic position and at 0.7 cM from *Lr.ace-4A-Eth* (to race EEEEE_Eth50-4) and 1.9 cM from *Lr.ace-4A-Tun* (to race BBBSJ_Tun20-4) (Figure 3). These small deviations in the mapping positions of the identified *Lr* gene region could be explained by the different numbers of RILs evaluated per isolate. Thus, it is most likely that resistance to these four *Pt* isolates was conferred by the same *Lr* gene designated as *Lr.ace-4A*. The tests for races BBBQJ_ Mor38-2 and BBBQJ_CA1.2 had the highest numbers of RILs evaluated (Table 2), therefore the mapping resolution should be higher for *Lr.ace-4A-CA* and *Lr.ace-4A-Mor. Lr.ace-4A* had LOD scores of 64.14, 74.64, 44.28, and 52.91 and accounted for 78, 81, 72, and 81% of phenotypic variations (R^2^) to the *Pt*-isolates BBBQJ_CA1.2, BBBQJ_ Mor38-2, EEEEE_Eth50-4, and BBBSJ_Tun20-4, respectively.

**Table 4:** Quantitative trait loci associated with stem rust resistance in the RIL population derived from Rusty × PI 192051-1

QTL *^a^*	Closest Marker (cM)	Closest SNP allele *^b^*	QTL Interval (cM) *^c^*	QTL Interval (bp) *^d^*	LOD (R^2^) *^e^*								
Trait *^f^*					QFCFC	TPMKC	TMLKC	JRCQC	TKTTF	TTKSK	Eth2017_1	Eth2017_2
*QSr.ace-1A*	*IWA8523* (141.2)	A/G	*IWA3409* – *IWA5734* (131.7 - 144.3)	571,580,052 – 593,399,125		3.03 (0.10)						
*QSr.ace-2B-a*	*IWA4890* (92.1)	A/G	*IWA2261* - *IWA6399* (90.9 - 92.7)	650,285,204 – 665,668,837				5.30 (0.13)				
*QSr.ace-2B-b*	*IWA6399* (92.7)	A/C	*IWA6399* - *IWA7955* (92.7 - 105.3)	665,668,638 – 720,480,388						5.98 (0.14)		
*QSr.ace-4A*	*IWA7521* (5.9)/ *IWA4657* (15.6) *^g^*	A/G / T/C	*IWA603* – *IWA4657* (0 – 15.6)	37,813,793 – 581470,783	3.11 (0.10)		3.56 (0.11)		3.06 (0.08)			
*QSr.ace-7A*	*IWA8390* (8.1)	T/C	*IWA8390* - *IWA1805* (8.1 - 9.6)	67,578,251 – 76,938,437	17.88 (0.45)	22.83 (0.58)	31.64 (0.67)	59.18 (0.78)	32.81 (0.6)	59.19 (0.79)	8.2 (0.21)	13.94 (0.33)

a All QTL were identified using composite interval mapping except for the QTL on 2B that were mapped using multiple interval mapping (MIM) and MIM-based on a general linearized framework (MIM-GLZ) for races TTKSK and JRCQC respectively.

b The underlined nucleotide is the SNP allele associated with stem rust resistance.

c 95% confidence intervals of the QTL were estimated using the 2-LOD drop method as described by Lander and Botstein (1989).

d Physical interval of the QTL based on the BLAST of flanking marker sequences against the genome sequence of the wheat cultivar Chinese Spring (RefSeq v1.0).

e LOD scores for each QTL are listed with the generalized R^2^ values in parenthesis.

f *Pgt* races QFCFC, TPMKC, TMLKC, JRCQC, TKTTF, and TTKSK were tested at seedling stage in the greenhouse. Eth2017_1, Eth2017_2 are adult plant stem rust phenotypes from two dates in Ethiopia 2017.

g *IWA7521* is the closest marker to the QTL peak to races QFCFC and TMLKC and *IWA4657* is the closest marker to the QTL peak TKTTF.

Eighty-seven SNPs representing 17 unique loci were mapped on the linkage group corresponding to chromosome 4A with a total length of 107.2 cM (Figure S2). The mapping region of *Lr.ace-4A* spanned the centromere with flanking markers *IWA232* (159,507,011 bp) and *IWA1793* (570,267,558 bp) on 4AS and 4AL, respectively based on BLAST of the SNP marker flanking sequences against the genome sequence of Chinese Spring wheat (RefSeq_v1.0) on the International Wheat Genome Sequencing Consortium (IWGS) website. Each marker flanking *Lr.ace-4A* had sets of co-segregating markers on either side of the centromere, where *IWA232* co-segregated with markers *IWA126*, *IWA8416*, *IWA4359*, *IWA6377*, *IWA4253*, and *IWA4254* and the SNP *IWA1793* co-segregated with *IWA8341* (Figure S2). The marker *IWA232* (11.9 cM) on 4AS was the closest to *Lr.ace-4A-CA*, *Lr.ace-4A-Mor*, and *Lr.ace-4A-Eth* therefore, *Lr.ace-4A* is probably located on chromosome 4AS very close to the centromere. The SNP *IWA232* allele “T’ is associated with leaf rust resistance while the marker allele ‘C’ is associated with susceptibility.

### QTL analysis for stem rust

**Figure 4:**
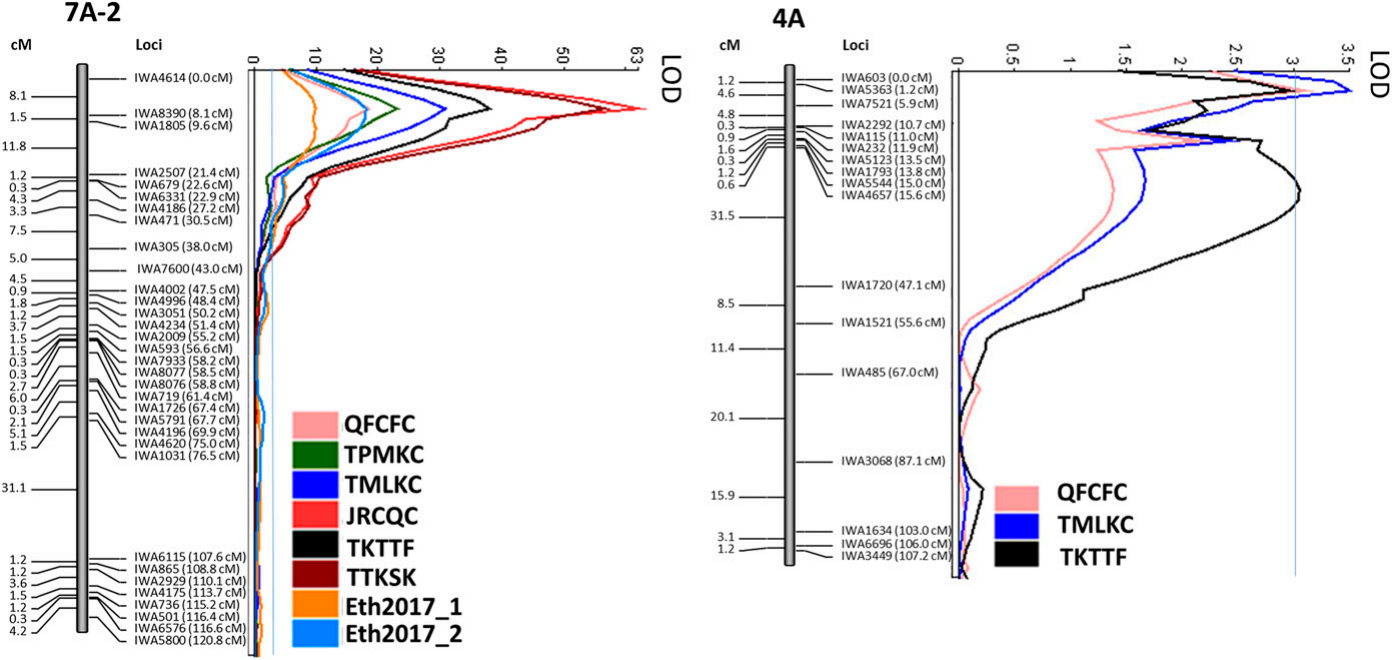
Mapping of stem rust resistance QTL (*QSr.ace-4A* and *QSr.ace-7A*) in the population Rusty × PI 192051-1 at seedling stage to races QFCFC, TPMKC, TMLKC, JRCQC, TKTTF, and TTKSK and at adult plant stage in stem rust nursery in Ethiopia in 2017. All QTL were identified using composite interval mapping. The QTL analysis LOD threshold is shown with the blue horizontal line. Co-segregating markers were excluded from these maps. SNPs and their cosegregating markers are presented in Figure S2.

The QTL analysis identified four stem rust resistance genes/QTL in PI 192051-1: *QSr.ace-7A*, *QSr.ace-4A*, *QSr.ace-1A*, and *QSr.ace-2B* localized on 7AS, 4A, 1AL, and 2BL, respectively. Linked SNPs to these stem rust resistance QTL and the SNP alleles associated with resistance were identified (Figure 4, Figure S3, and Table 4). *QSr.ace-7A* conferring resistance to all six *Pgt* races tested at the seedling stage and to stem rust at adult plant stage in Eth2017_1 and Eth2017_2 was identified using CIM. Sixty-eight SNPs, representing 33 unique loci were mapped to linkage group 7A-2 covering 120.80 cM (Figure S2). *QSr.ace-7A* was mapped to a 1.5 cM region flanked by *IWA8390* and *IWA1805*, with *IWA8390* (8.1 cM) being the closest marker to this QTL. Based on the physical positions of the QTL flanking markers, *QSr.ace-7A* interval was within 67,578,251 – 76,938,437 bp. *QSr.ace-7A* had LOD values of 17.88, 22.83, 31.64, 59.18, 32.81 and 59.19 and explained 45, 58, 67, 78, 60, and 79% of disease variation to *Pgt* races QFCFC, TPMKC, TMLKC, JRCQC, TKTTF and TTKSK, respectively. In Ethiopia, *QSr.ace-7A* was identified at both scoring dates with LOD values of 8.20 and 13.94 and R^2^ of 21 and 33% for Eth2017_1 and Eth2017_2, respectively (Figure 4 and Table 4).

RILs with the susceptibility ‘T’ allele of *QSr.ace-7A* closest marker *IWA8390* had average stem rust IT scores of 7.7, 7.6, 7.9, 8.8, 8.9, and 8.6 to *Pgt*-races QFCFC, TPMKC, TMLKC, JRCQC, TKTTF and TTKSK, respectively and an average stem rust severity of 50% in the field test Eth2017_2. The RILs with the resistance ‘C’ allele of the SNP *IWA1805* had average stem rust IT scores of 5.3, 5.3, 5.4, 5.3, 6.1, and 4.8 to *Pgt*-races QFCFC, TPMKC, TMLKC, JRCQC, TKTTF and TTKSK, respectively, and an average stem rust severity of 31.3% in field test Eth2017_2. The distribution of RILs based on alleles at *IWA8390* had little overlap. For instance only four RILs with the *IWA8390* susceptibility ‘T’ allele from Rusty had stem rust IT scores < 6 and none of the RILs with the resistance allele from PI 192051-1 had a stem rust IT score > 6. In the field test Eth2017_2 the distribution of RILs based on alleles at *IWA8390* also had little overlap where only three RILs with the resistance allele had severities >50% and only five RILs with the *IWA8390* susceptibility allele had severities less than 30%. Very similar results were recorded for the other *QSr.ace-7A* flanking marker *IWA1805* (Figure S4).

A minor effect stem rust QTL, designated *QSr.ace-4A*, was identified using CIM on the chromosome 4A (Figure 4 and Table 4). *QSr.ace-4A* had LOD values of 3.11, 3.56, and 3.06 and explained 10, 11, and 8% of the variation in stem rust response caused by races QFCFC, TMLKC, and TKTTF respectively. It was flanked by markers *IWA603* and *IWA4657* (0 – 15.6 cM), with *IWA7521* (5.9 cM) being the closest marker to the QTL peak for races QFCFC and TMLKC, and *IWA4657* on chromosome 4AL being the closest marker to the QTL peak for race TKTTF. Markers *IWA568*, *IWA4431*, *IWA482*, *IWA4432*, *IWA483*, and *IWA569* co-segregated with *IWA4657* (Figure S2). *QSr.ace-4A* spanned the centromere with flanking markers *IWA603* and *IWA4657* located on chromosomes 4AS and 4AL, respectively. Based on the physical positions of the QTL flanking markers, *QSr.ace-4A* interval was within 37,813,793 – 581,470,783 bp (Figure 4 and Table 4).

Epistatic interactions were investigated by grouping RILs based on their *QSr.ace.7A*and *QSr.ace-4A* genotypes and statistically comparing the disease means of each RIL group to QFCFC, TMLKC, and TKTTF using Tukey’s honest significant difference test (HSD) (Tukey 1949) at 95% level of confidence (Table 5). The RILs with both *QSr.ace-7A* and *QSr.ace-4A* had lower linearized stem rust ITs compared to that of the RIL with only *QSr.ace-7A*, however this difference was statistically significant only for race TKTTF based on Tukey’s test (*P* < 0.05). This suggests that *QSr.ace-4A* enhances *QSr.ace.7A* resistance to *Pgt* race TKTTF. The infection types of the RILs carrying only *QSr.ace-4A* and those with neither QTL were not significantly different (Table 5).

**Table 5:** Epistatic interactions of stem rust QTL in Rusty × PI 192051-1 population

Race	QTL *^a^*	RIL group	Number of RILs	Mean of disease *^b^*	Std Dev	Tukey’s test *^c^*
QFCFC	*QSr.ace-4A*, *QSr.ace-7A*	*QSr.ace-4A* & *QSr.ace-7A*	51	5.11	0.91	A
		*QSr.ace-7A*	29	5.55	1.21	A
		*QSr.ace-4A*	12	7.75	0.97	B
		None	30	7.63	0.93	B
TMLKC	*QSr.ace-4A*, *QSr.ace-7A*	*QSr.ace-4A* & *QSr.ace-7A*	50	5.18	0.65	A
		*QSr.ace-7A*	30	5.38	0.88	A
		*QSr.ace-4A*	14	7.79	0.8	B
		None	32	7.88	0.71	B
TKTTF	*QSr.ace-4A*, *QSr.ace-7A*	*QSr.ace-4A* & *QSr.ace-7A*	57	5.90	0.95	A
		*QSr.ace-7A*	42	6.40	1.03	B
		*QSr.ace-4A*	21	8.71	0.12	C
		None	33	8.82	0.40	C
TPMKC	*QSr.ace-1A*, *QSr.ace-7A*	*QSr.ace-1A* & *QSr.ace-7A*	41	4.96	0.13	A
		*QSr.ace-7A*	38	5.49	1.21	B
		*QSr.ace-1A*	14	7.36	1.15	C
		None	25	7.76	0.83	C
JRCQC	*QSr.ace-2B-a*, *QSr.ace-7A*	*QSr.ace-2B-a* & *QSr.ace-7A*	57	5.21	0.58	A
		*QSr.ace-7A*	46	5.43	0.86	A
		*QSr.ace-2B-a*	28	8.69	0.89	B
		None	25	8.79	0.43	B
TTKSK	*QSr.ace-2B-b*, *QSr.ace-7A*	*QSr.ace-2B-b* & *QSr.ace-7A*	50	4.35	0.27	A
		*QSr.ace-7A*	47	4.56	0.4	A
		*QSr.ace-2B-b*	26	8.26	1.12	B
		None	31	8.27	1.02	B

a All QTL were identified using composite interval mapping except for the QTL on 2B that were mapped using multiple interval mapping (MIM) and MIM-based on a general linearized framework (MIM-GLZ) for race TTKSK and JRCQC respectively.

b Disease scores were based on a linearized Stakman scale (0-9) for all races.

c Numbers in the same column followed by the same letter are not significantly different at *P* = 0.05.

Minor QTL *QSr.ace-1A* conferring resistance to *Pgt* race TPMKC on the distal end of the chromosome 1AL was identified using CIM. Ninety-four SNPs, representing 34 unique loci spanned 144.3 cM. *QSr.ace-1A* peaking between markers *IWA3409* and *IWA5734* (131.7 – 144.3 cM) with a LOD score of 3.03 explained 10% of the variation in stem rust response (Figure S3 and Table 4). Based on the physical positions of the flanking markers, the *QSr.ace-1A* physical confidence interval was within the region 571,580,052 – 593,399,125 bp. *IWA8523* (141.2 cM) was the closest marker to *QSr.ace-1A. IWA3409* co-segregated with *IW2450* and *IWA5734* co-segregated with *IWA3799*, *IWA3492*, and *IWA5806* (Figure S2). RILs with both *QSr.ace-1A* and *QSr.ace-7A* had significantly lower stem rust ITs compared to RILs with only *QSr.ace-7A* based on Tukey’s test (*P <* 0.05*)*. This suggests that *QSr.ace-1A* enhances *QSr.ace.7A* resistance to *Pgt* race TPMKC. The presence of only *QSr.ace-1A* did not confer detectable resistance to race TPMKC (Table 5).

Even though the segregation ratio of the RILs to *Pgt* races TTKSK and JRCQC did not fit a single gene model based on χ2 test at 95% level of confidence, no additional QTL to *QSr.ace.7A* was identified using CIM. Therefore, other QTL mapping methods MIM and MIM-GLZ were used to investigate the possibility of other loci being involved in resistance to these races. The MIM-GLZ algorithm is known to have greater power to overcome model assumption violations in phenotypic data compared to traditional algorithms such as CIM and MIM that rely on transformed phenotypic data (Joehanes 2009; Dahleen *et al.* 2012; Zurn *et al.* 2018). The use of MIM-GLZ helped to detect *QSr.ace-2B-a* to race JRCQC flanked by *IWA2261* and *IWA6399* (90.9 – 92.7) at the distal end of chromosome 2BL. BLAST of the flanking SNP sequences against the Chinese Spring genome sequence showed that *QSr.ace-2B-a* was within 650,285,204 – 665,668,837 bp. *QSr.ace-2B-a* had a LOD score of 5.30 (LOD threshold = 4.79) and explained 13% of the phenotypic variation. MIM revealed identified *QSr.ace-2B-b* to race TTKSK flanked by *IWA6399* and *IWA7955* (92.7 – 105.3) on chromosome 2BL. The physical positions of the flanking markers showed that *QSr.ace-2B-b* was within 665,668,638 – 720,480,388 bp. This QTL with a LOD score of 5.98 (LOD threshold = 3.72) explained 14% of the phenotypic variation. Markers flanking both QTL also had co-segregating markers (Figure S2). Markers *IWA469/IWA5414* (92.2 cM) and *IWA2318/IWA6399* (92.7 cM) were important in detecting *QSr.ace-2B-a*, whereas *IWA5414* (92.2 cM) and *IWA6399* (92.7 cM) were necessary in mapping *QSr.ace-2B-b*. The genomic and physical regions of both *QSr.ace-2B-a* and *QSr.ace-2B-b* overlapped suggesting they were likely same QTL conferring resistance to races TTKSK and JRCQC. These QTL are henceforth designated *QSr.ace-2B* (Figure S3 and Table 4). RILs with both *QSr.ace-2B* and *QSr.ace-7A* had lower linearized stem rust infection types (4.35 to TTKSK and 5.21 to JRCQC) compared to those of other RIL genotypes, however, this difference was not significantly lower than that of RILs genotypes carrying *QSr.ace-7A* alone based on Tukey’s test at 95% level of confidence (Table 5).

## Discussion

New sources of resistance are essential to effectively protect durum production against continuously and rapidly evolving rust pathogens. In the current study PI 192051-1 showed resistance to durum type-*Pt* isolates collected in Ethiopia, Morocco, USA, and Tunisia. This genotype also carries stem rust resistance that is effective against both African and North American *Pgt* races. A *Lr* gene *Lr.ace-4A*, and four *Sr* QTL, *QSr.ace-7A*, *QSr.ace-1A*, *QSr.ace-2B* and *QSr.ace-4A*, were identified in PI 192051-1.Linked SNPs to these stem rust resistance QTL and leaf rust resistance gene and the SNP alleles associated with resistance were identified. Primer sequences for development of Kompetitive allele specific PCR (KASP) markers for all wheat Illumina SNPs, including those identified as associated with resistance in this study, are publicly available at http://polymarker.tgac.ac.uk/ (Ramirez-Gonzalez *et al.* 2014, 2015).

*Lr.ace-4A* conferring resistance at the seedling stage was mapped to the centromere region of chromosome 4AS. The only previously cataloged *Lr* gene on chromosome 4AL and close to the centromere is *Lr30*, identified in common wheat cultivar Terenzio (Dyck and Kerber 1981). To our knowledge, *Lr30* has not been reported in durum and also appears to be quite rare in common wheat germplasm. For instance, a *Lr* gene postulation study on a worldwide common wheat collection of 275 accessions showed that only two accessions from North America possibly carried *Lr30* (Dakouri *et al.* 2013). An evaluation of PI 192051-1 with North American common wheat *Pt*- race TNRJJ that is virulent on Thatcher NIL carrying *Lr30* showed that PI 192051-1 was highly resistant (IT ;) (M. Aoun, unpublished). However, PI 192051-1 could have *Lr72* or another durum gene that gives resistance to common type wheat races. Based on the *Lr30* map on the GrainGenes website (https://wheat.pw.usda.gov/GG3/), this gene is flanked by *IWA4359* (physical position: 531,679,637 bp) and *IWA2585* (physical position: 544,177,878 bp). *IWA4359* is one of the flanking makers of both *Lr.ace-4A and Lr30*. There was an overlap of the physical regions of the durum gene *Lr.ace-4A* and the common wheat gene *Lr30*. *Lr30* was reported as a recessive resistance gene (Dyck and Kerber 1981) which suggests it may be different from the dominant resistant gene *Lr.ace-4A* in PI 192051-1. However, it is possible that expression differs in a durum background compared to a common wheat background. There are no reports of cataloged *Lr* genes in durum wheat on chromosome 4A thus, *Lr.ace-4A* is considered a novel *Lr* gene in durum.

Markers associated with leaf rust resistance on 4A were observed in an association mapping study (AM) using the USDA-NSGC from which PI 192051 was selected (Aoun *et al.* 2016). The AM revealed five SNPs associated with leaf rust response on 4A. One of these was *IWA1570* (59.9 cM) within the mapped region of *Lr.ace-4A* (51.3–64.0 cM) based on the tetraploid wheat consensus map (Maccaferri *et al.* 2015) and marker physical positions on the Chinese Spring genome sequence. PI 192051-1 is resistant to several durum type *Pt* isolates virulent to *Lr3a*, *Lr27+31*, *Lr61*, and *Lr72* (Aoun *et al.* 2016). This Portuguese landrace showed a high level of adult-plant stage resistance in field trials conducted in USA, Mexico (Aoun *et al.* 2016), Morocco, and Ethiopia (Aoun and Acevedo, unpublished). Therefore, future evaluation of the Rusty × PI 192051-1 population to other *Pt* isolates at seedling and at adult plant stages in several geographical locations could reveal other genes in PI 192051-1. One of the *Pt*-isolates used in this study, BBBSJ_Tun20-4 is virulent to the widely used *Lr14a*, therefore the deployment *Lr.ace-4A* in breeding programs could provide an additional source of resistance particularly in the regions where virulence to *Lr14a* is prevalent (Ordoñez and Kolmer. 2007a; Goyeau *et al.* 2006; Soleiman *et al.* 2016).

PI 192051-1 also carries *QSr.ace-7A* on chromosome arm 7AS conferring seedling resistance to both North American and African *Pgt*-races as well as to *Pgt*-races at the adult-plant stage in Eth2017. Chao *et al.* (2017) reported that PI 192051 was resistant at seedling stage to many USA *Pgt* races, including BCCBC, MCCFC, QFCSC, QTHJC, RCRSC, RKQQC, and TTTTF, but was susceptible to the Ethiopian race TRTTF. The same study showed a high resistance level in PI 1902051 at the adult plant stage in stem rust nurseries at St. Paul, USA and Debre Zeit, in Ethiopia. Since cataloged *Sr* genes on chromosome 7AS have not been characterized in tetraploid or hexaploid wheat, *QSr.ace-7A* is a novel *Sr* gene in wheat. A number of QTL on 7A, associated with stem rust resistance at both the seedling and adult-plant stages have been reported in AM and linkage mapping studies in durum wheat (Haile *et al.* 2012; Letta *et al.* 2013; 2014; Chao *et al.* 2017). Chao *et al.* (2017) showed that *IWA7200* was associated with stem rust response in the durum lines in the USDA-NSGC. The SNP *IWA7200* (68.7 cM) on 7AS is close to the mapped position of *QSr.ace-7A* (*IWA8390* at 62.4 cM), based on the tetraploid wheat consensus map (Maccaferri *et al.* 2015) and also based on the physical positions of the markers on the Chinese Spring genome sequence. Letta *et al.* (2013, 2014) reported two DArt markers on 7AS (*wPt-2799* and *wPt-7885)* associated with response to *Pgt*-races in field trials in Ethiopia in a durum panel of 183 cultivars and breeding lines. In another study in Ethiopia Haile *et al.* (2012) identified QTL for stem rust resistance on chromosome 7A in the durum cultivar Sebatel. One of the QTL on 7AS (*QSr.1PK-7A.1*) was flanked by SSR markers *gwm974* and *gwm631*, while the second (*QSr.1PK-7A.2*) was thought to be *Sr22* which is located on 7AL. However, *Sr22* is a diploid wheat-derived gene and its presence in durum would be unlikely. Since the markers flanking *QSr.1PK-7A.1* are absent in the tetraploid consensus map and the SSR flanking sequence information is not available on the GrainGenes database, comparison between the genomic locations of *QSr.ace-7A* and *QSr.1PK-7A.1* was not possible.

Three minor stem rust resistance QTL were inherited from PI 192051-1 (*QSr.ace-1A*, *QSr.ace-2B* and *QSr.ace-4A*). *QSr.ace-2B* is close to the position of *Sr9* (Rouse *et al.* 2014a). Since JRCQC and TTKSK are virulent to *Sr9e*, *QSr.ace-2B* could be *Sr9h* that is effective against race TTKSK and originally from durum cv. Gaza (Nirmala *et al.* 2016). In an AM study by Letta *et al.* (2014) *gwm1300* was associated with seedling resistance to races TTTTF and TTKSK. This SSR marker is very close to the position of *QSr.ace-2B* based on marker positions in the tetraploid consensus map.

In this study we observed an overlap between the genomic regions of *QSr.ace-4A* (0 – 15.9 cM) and *Lr.ace-4A* (11.9 – 15.9 cM) and a significant correlation between stem rust responses and leaf rust responses of the RILs (r= 0.2, *P* ≤ 0.05). This suggests *Lr.ace-4A* is co-located or linked to a stem rust resistance gene with minor effect (*QSr.ace-4A*) to races QFCFC, TMLKC and TKTTF. The *Sr* genes *Sr7* (Knott 1959, Knott and Anderson 1956, McIntosh *et al.* 1995) and *SrND643* thought to be an allele of *Sr7* (Basnet *et al.* 2015) are present on chromosome 4AL. Saini *et al.* (2018) reported *QSr.rwg-4A* (likely to be *Sr7a*) in the durum cultivar Lebsock at the distal end of chromosome 4AL whereas *QSr.ace-4A* was located in the pericentromeric region of chromosome 4A, thus *QSr.ace-4A* is unlikely to be *Sr7*.

*QSr.ace-1A* on chromosome 1AL was associated with resistance to *Pgt*-race TPMKC at the seedling stage. A cataloged *Sr* gene mapped to chromosome 1AL was not previously detected; however, *QSr.ipk-1* conferring stem rust resistance at the adult plant stage in the durum cultivar Sebatel was located on 1AL (Haile *et al.* 2012). The SSR markers *Xbarc148* and *Xbarc119* flanking *QSr.ipk-1* are distant from the position of *QSr.ace-1A* based on marker locations in the tetraploid consensus map and the marker physical positions on the Chinese Spring genome sequence. Rouse *et al.* (2014b) mapped *QSr.cdl-1AL*, an adult plant resistance QTL, at the distal end of chromosome1AL in Thatcher, a derivative of Iumillo durum. *QSr.cdl-1AL* was tightly linked to the DArt marker *XwPt6869* which was associated with epistatic interactions for leaf rust resistance in durum wheat (Singh *et al.* 2013).

In this study, we identified significant positive (enhanced resistance) QTL interactions. The two minor effect QTL, *QSr.ace-4A* and *QSr.ace-1A*, enhanced significantly the seedling resistance of *QSr.ace-7A* to *Pgt* races TKTTF and TPMKC, respectively. However, these QTL does not provide resistance in the absence of *QSr.ace-7A*. This implies that breeders will not grain much by introgressing only the minor QTL, thus *QSr.ace-4A* and *QSr.ace-1A* will be useful only if accompanied by *QSr.ace-7A*.

This study revealed a number of stem rust and leaf rust resistance genes/QTL with major (*Lr.ace-4A* and *QSr.ace-7A*) and minor effects (*QSr.ace-1A*, *QSr.ace-2B*, and *QSr.ace-4A*) in durum landrace PI 192051-1. This is the first study to identify a *Lr* gene on chromosome 4A (*Lr.ace-4A*) of durum wheat. Stem rust resistance QTL *QSr.ace-2B* on chromosome 2BS is possibly *Sr9h*, and the remainder QTL (*QSr.ace-1A*, *QSr.ace-4A*, and *QSr.ace-7A*) appear to be uncharacterized. *QSr.ace-7A* was mapped on chromosome arm 7AS where no previously cataloged *Sr* gene has been identified in either durum or common wheat. *Lr.ace4A* could be the same or closely linked to a minor QTL for stem rust resistance named *QSr.ace-4A*. Genes/QTL that confer resistance to multiple diseases are suggestive of new molecular structures, as well as allowing simultaneous breeding. Three of the resistance genes/QTL in PI 192051-1 (*Lr.ace-4A*, *QSr.ace-4A*, and *QSr.ace-7A*) were effective against diverse *Pt* or *Pgt* races from different countries. Markers closely linked to these QTL will facilitate their introgression into adapted durum wheat cultivars.
